# Subclinical systolic and diastolic dysfunction in women with signs and symptoms of ischemia but no obstructive coronary disease: novel insights using myocardial feature tracking in the NHLBI WISE study

**DOI:** 10.1186/1532-429X-18-S1-O3

**Published:** 2016-01-27

**Authors:** Michael D Nelson, Jaime L Shaw, Janet Wei, Chrisandra Shufelt, Puja K Mehta, Manish Motwani, Louise E Thomson, Daniel S Berman, Richard B Thompson, Debiao Li, C Noel Bairey Merz, Behzad Sharif

**Affiliations:** 1 Biomedical Imaging Research Institute, Cedars-Sinai Medical Center, Los Angeles, CA USA; 2 Barbra Streisand Women's Heart Center, Cedars-Sinai Medical Center, Los Angeles, CA USA; 3 Cedars-Sinai Heart Institute, Los Angeles, CA USA; 4 Department of Biomedical Engineering, University of Alberta, Edmonton, AB Canada

## Background

Women with signs and symptoms of ischemia--but no obstructive coronary artery disease--often have coronary microvascular dysfunction (CMD), and are at increased risk of major cardiovascular events, including heart failure. Using cardiac magnetic resonance tissue tagging, we recently found subclinical diastolic dysfunction in these women, suggesting that ischemia-related diastolic dysfunction may be mechanistically linked to the development of heart failure. Tissue tagging involves specialized image acquisition and post processing, and thus is not typically suitable for large patient studies. We hypothesized that feature tracking of conventional (clinical) cardiac cine images would provide similar information. We therefore compared feature tracking with gold-standard tissue tagging, and then, used feature tracking to assess left ventricular function in women with suspected CMD.

## Methods

We first conducted a validation study comparing feature tracking (applied to standard cine images) to the gold-standard tissue tagging approach (applied to tagged cine images). To this end, in n = 59 individuals, we measured circumferential strain and diastolic circumferential strain rate (CSdR) by tissue tagging (HARP, Diagnosoft) and feature tracking (cvi42, Circle Cardiovascular Imaging). Following the validation study, we performed feature tracking on cine images from n = 116 women with suspected CMD ("cases") enrolled in the NHLBI-sponsored Women's Ischemia Syndrome Evaluation (WISE) Study, and n = 28 aged-matched healthy reference controls.

## Results

**Figure 1:**
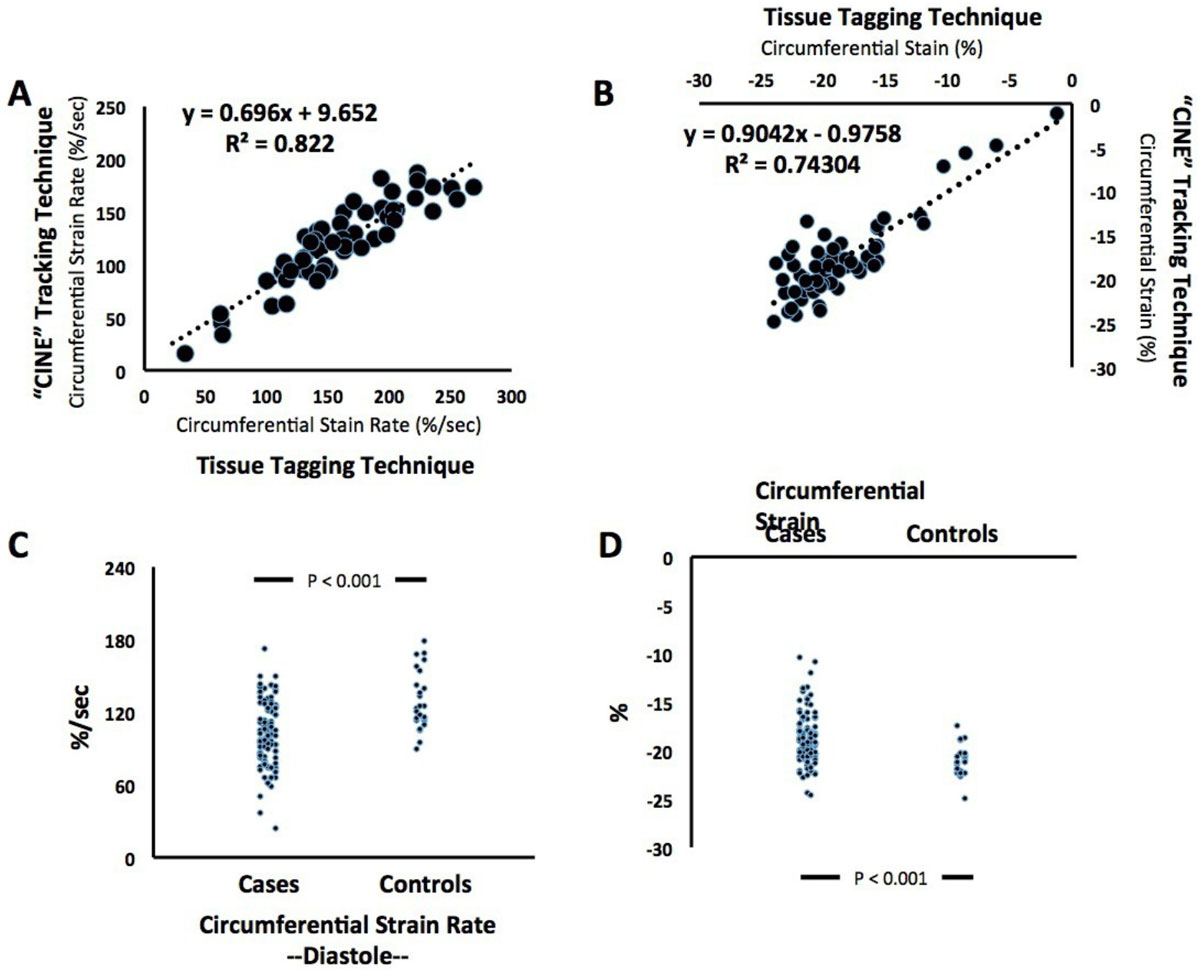
**Feature tracking is highly correlated with gold-standard MR tissue tagging, for both diastolic circumferential strain rate (Panel A) and systolic circumferential strain (Panel B), therefore validating its applicability for myocardial function analysis**. Application of this technique reveals significant differences in both diastolic (Panel **C**) and systolic function (Panel **D**) between women with suspected coronary microvascular dysfunction (n = 116 cases) and aged-matched healthy controls (n = 28).

In the validation study, feature tracking correlated with tissue tagging (Fig. 1A-B), providing similar results for CSdR and circumferential strain (P < 0.01 for both). Our case-control comparison: (a) confirmed previous observations in the WISE cohort, demonstrating reduced CSdR in cases with suspected CMD compared to controls (Fig. 1C); and (b) revealed subclinical systolic dysfunction, as circumferential strain was impaired in cases compared to controls (Fig. 1D).

## Conclusions

Feature tracking applied to cine myocardial function images provides a convenient method to analyze wall motion in conventional CMR protocols. In this study, we used this technique to both confirm and extend previous observations in a cohort of women with suspected CMD, demonstrating subclinical diastolic and systolic dysfunction. Further analysis is warranted to better characterize the advantages and limitations of this approach in studies involving patients with suspected CMD and no obstructive disease.

